# Safety of the “Saxophone^®^” electrode in parotid surgery for continuous intraoperative neuromonitoring of the facial nerve: results of a pro- and retrospective cohort study

**DOI:** 10.1007/s00405-020-05803-4

**Published:** 2020-01-27

**Authors:** Petar Stankovic, Jan Wittlinger, Robert Georgiew, Nina Dominas, Katrin Reimann, Stephan Hoch, Thomas Wilhelm, Thomas Günzel

**Affiliations:** 1 Department of Otolaryngology, Head/Neck and Facial Plastic Surgery, Sana Kliniken Leipziger Land, Rudolf-Virchow-Strasse 2, 04552 Borna, Germany; 2 Department of Otolaryngology, Head/Neck and Facial Plastic Surgery, Martin Luther University of Halle-Wittenberg, Wittenberg, Germany; 3 Department of Otolaryngology, Head/Neck and Facial Plastic Surgery, Philipps University of Marburg, Marburg, Germany; 4 Medical Faculty, Philipps University of Marburg, Marburg, Germany; 5 Department of Otolaryngology, Head and Neck Surgery, Borromaeus Hospital Leer, Leer, Germany

**Keywords:** cIONM, Intraoperative neuromonitoring, Parotidectomy, Facial palsy

## Abstract

**Purpose:**

Early facial nerve palsy (eFNP) is the most frequent complication of the parotidectomy. Intraoperative neuromonitoring (IONM) in parotid surgery, which aims at reducing eFNP, has not evolved any further than the mere differentiation between the nerve and the surrounding tissue. Continuous IONM (cIONM), used in thyroid and posterior fossa surgery, has developed over the past years and has proved beneficial in reducing the rate of paresis in cases where a pattern of impending nerve injury is identified. In this study, we aim to demonstrate the safety of using the stimulating electrode (Saxophone^®^) for cIONM in parotid surgery.

**Methods:**

From 2016 to 2018, 40 patients who were referred for primary parotidectomy under cIONM according to our study protocol (registered at the German Clinical Trials Register, DRKS-ID: DRKS00011051, http://www.drks.de; http://apps.who.int/trialsearch) were included in this study. All patients with a normal preoperative facial nerve function [House–Brackman (HB)-Index 1] underwent surgery using continuous facial nerve stimulation with the Saxophone^®^ electrode (system AVALANCHE XT, Dr. Langer Medical, Waldkirch, Germany). A control group which underwent parotidectomies with only intermittent IONM was recruited from our records.

**Results:**

Half of the patients in our study group suffered from eFNP. All except one regained normal facial nerve function within 6 months of surgery. There was no significant difference regarding eFNP when compared to the control group without cIONM (*p* = 0.11). No statistically significant correlation between the stimulation threshold (*p* = 0.74) or the duration of nerve stimulation and eFNP was found (*p* = 0.51).

**Conclusion:**

We have demonstrated the safety of using the Saxophone^®^-electrode for cIONM of the facial nerve in parotid surgery. Future development of this method could enable the recognition of impending nerve injury and thus reduce eFNP.

## Introduction

Early facial nerve palsy (eFNP) is the most frequent complication of parotidectomy with reported rates of 28.8–77.2% in primary surgery [1–5]. This pertains to superficial lateral or partial superficial lateral parotidectomy with nerve dissection. It has been shown that eFNP has a significant impact on the physical and psychosocial well-being of the patient [5].

In many institutions, efforts have been made to reduce the rate of eFNP by making use of intraoperative neuromonitoring (IONM). A recent meta-analysis showed IONM to significantly reduce eFNP [6]. Although the history of IONM started in 1969 [7], no significant breakthrough in the method has been noted and consequently IONM in parotid surgery is used merely to differentiate between the nerve and the surrounding tissue.

Novel methods of continuous IONM (cIONM) in which the vagus and the facial nerve are continuously stimulated to acquire “real-time” information of the nerve function have evolved in other surgical procedures, though mainly in thyroidectomy and surgery on the posterior cranial fossa. Patterns of impending nerve injury are thereby identifiable, providing a signal to the surgeon who is then able to modify the causative surgical movement and thus avoid nerve injury [8, 9].

Inspired by these findings, we have aimed to implement this method in parotid surgery, seek out a pattern of nerve injury and integrate this pattern into an algorithm which will then warn the surgeon of impending nerve injury.

## Material and methods

A prospective, non-randomized study was carried out and the results thereof compared to a retrospective cohort from our department. All patients enrolled in the study provided informed written consent. Ethical approval of the study protocol was obtained from the Saxonian Chamber of Physicians, Germany (25 August 2016, EK-BR-53/16-1). The study was registered at the German Clinical Trials Register prior to conducting the research (ID: DRKS00011051, http://www.drks.de; http://apps.who.int/trialsearch). From October 2016 to October 2018, all parotid surgery patients admitted to our tertiary care hospital were checked for their potential eligibility to enroll in this study.

Inclusion criteria were: preoperative intact function of the facial nerve on both sides [according to House–Brackmann (HB)-Grade 1 [10]], no previous parotid surgery, signed informed consent, and an age of over 18 years. The exclusion criteria were preoperative facial paresis (HB ≥ 2), revision surgery, an age of under 18 years, total parotidectomy with resection of the facial nerve, pace-maker or defibrillator, pregnancy, epilepsy, and heart rhythm problems. The evaluation of the facial nerve function was obtained preoperatively from two independent clinicians and photo documentation.

All surgery was performed under general anesthesia. Prior to skin incision, a pair of monopolar needles were placed in the facial musculature in order to provide four channel EMG-neuromonitoring (frontalis, orbicularis oculi, nasalis, and orbicularis oris muscle; neuromonitoring System: Avalanche^®^ XT [Software Avalanche^®^ SI], Dr. Langer Medical, Waldkirch/Germany).

**Fig. 1:**
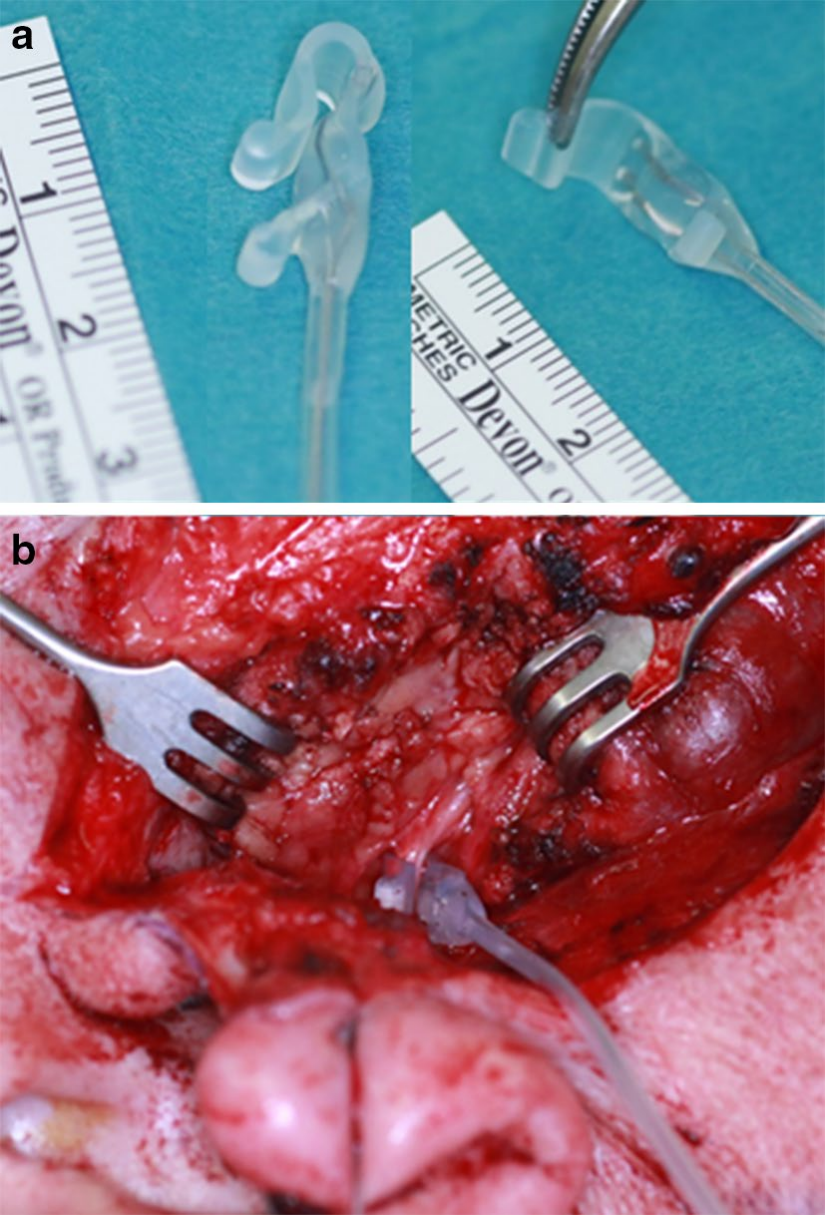
The Saxophone^®^ electrode (**a**) for continuous stimulation used on the facial nerve trunk in a right parotidectomy (**b**)

The parotidectomies were performed in the usual anterograde dissection manner after performing the Blair skin incision. Three consultant surgeons operated on all patients using binocular loupes as an optical aid. After identifying the facial nerve trunk, the entire trunk was exposed in every direction by careful preparation with an atraumatic instrument. The Saxophone^®^ electrode (Fig. 1a, Dr. Langer Medical, Waldkirch/Germany) was then placed on the facial nerve trunk (Fig. 1b). The current threshold was then gradually calibrated in 0.1 mA increments, beginning at 0.1 mA, but never exceeding 1 mA, until the amplitudes of the four channels reached maximum values. The nerve trunk was then continuously stimulated by the calibrated current during the preparation of the nerve and the removal of the tumour at a frequency of 3 Hz and a pulse width of 200 µs. The moment the tumour was removed, the Saxophone^®^ electrode was detached. All information obtained during the operation, that is the amplitudes and latencies of the four channels, was automatically recorded in the neuromonitoring device. The facial nerve function was evaluated on the first postoperative day as well as 1 month and six months postoperatively by three independent observers.

Statistical analysis was performed using MedCalc for Windows, version 18.11.3 (MedCalc Software, Ostend/Belgium). To compare the early paresis rate in our prospective study to patients who had undergone surgery without the Saxophone^®^ electrode, we retrospectively analyzed the last 40 patients prior to the study who underwent a parotidectomy with intermittent IONM (iIONM,) using an electrode, applying the same inclusion and exclusion criteria. Fisher’s exact test was applied to calculate statistical significance. Correlation was calculated according to Pearson’s correlation coefficient *r* and presented with a 95% confidence interval. A *p* value of < 0.05 was set for statistical significance in all of the above-mentioned methods.

## Results

Between October 2016 and October 2018, 51 parotidectomies were performed. 11 patients were excluded from the study. The reasons for exclusion were as follows: implanted defibrillator, rejected taking part in the study and signing informed consent, intraoperative sacrifice of the facial nerve due to malignant involvement (each excluding two patients); fibrosis of the facial nerve trunk, pseudocholinesterase deficiency, revision operation, preoperative facial nerve palsy, and no identification of the facial nerve trunk (each excluding one patient). The facial nerve was stimulated supramaximal with a threshold of 0.62 ± 0.17 mA for 37.4 ± 16.7 min. The placement time of the Saxophone^®^ electrode was 257 ± 152.3 s while the electrode was displaced 1.4 ± 1.9 times. The postoperative histology showed a benign tumour in 37 (92.5%) cases. The average tumour volume was 9.7 ± 15.7 ml (Table 1).

**Table 1:** Demography, histology, duration of facial nerve stimulation and stimulation threshold of the patients in the cIONM-group

	cIONM		iIONM		*p*
	*N*	%	*N*	%	
Sex					
Male	14	35	23	57.5	
Female	26	65	17	42.5	0.07
Right side	18	45	22	55	0.5
Histology					0.54
Cystadenoma lymphomatosum (Warthin’s tumor)	16	40	19	47.5	
Pleomorphic adenoma	12	30	7	17.5	
Ruptured salivary duct	4	10	4	10	
Basal cell adenoma	–	–	3	7.5	
Lymh node	2	5	1	2.5	
Lymphoepithelial cyst	1	2.5	1	2.5	
Canalicular adenoma	1	2.5	–	–	
Sialolithiasis	1	2.5	–	–	
Oncocytoma	–	–	1	2.5	
Adenocarcinoma	1	2.5	1	2.5	
Acinar cell carcinoma	1	2.5	–	–	
Epithelial-myoepithelial carcinoma	1	2.5	–	–	
Mucoepidermoid carcinoma	–	–	1	2.5	
Metastasis of adenocarcinoma	–	–	1	2.5	
Metastasis of melanoma	–	–	1	2.5	
Benign tumor	37	92.5	36	90	0.69
Malignant tumor	3	7.5	4	10	
Tumor volume (ml)	9.7 ± 15.7		9.8 ± 13.2		0.52
Duration of nerve stimulation (min)	37.4 ± 16.7			–	
Stimulation threshold (mA)	0.62 ± 0.17			–	
Electrode implantation time (s)	257 ± 152.3			–	
Electrode dislocation	1.4 ± 1.9			–	

**Fig. 2:**
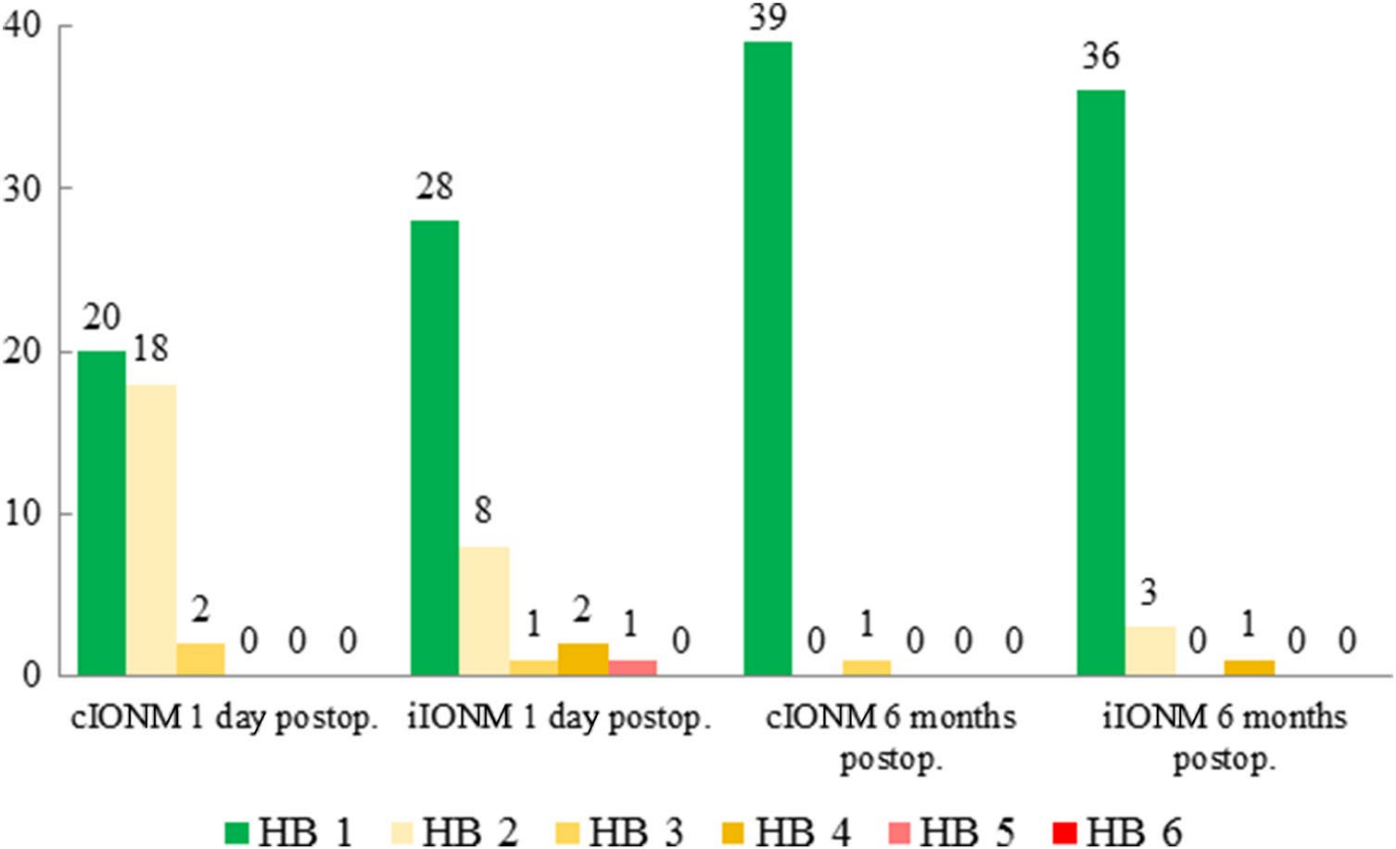
Postoperative facial nerve function according to House–Brackmann (HB) in 40 prospective patients undergoing surgery using the Saxophone^®^ electrode (cIONM) and the last 40 patients undergoing surgery without the electrode (iIONM) prior to the prospective study

The facial nerve function on the first postoperative day was as follows: 20 patients (50%) with HB 1, 18 patients (45%) with HB 2 and 2 patients (5%) with HB 3. No patients exhibited decreased facial nerve function (HB 4–5). One month postoperatively, 29 patients displayed an HB-grade 1 (72.5%), 10 patients an HB 2 (25%) and 1 patient an HB 3 (2.5%). Six months postoperatively, all but 1 patient improved to HB-grade 1 (39 patients HB 1–97.5%; 1 patient HB 3–2.5%; Fig. 2).

When one compared the last 40 parotidectomies performed prior to this prospective study without the Saxophone^®^ electrode (iIONM) to this cohort, using the same inclusion and exclusion criteria, no statistically significant difference was found with regard to facial nerve function (Table 2, *p* = 0.11) on the first postoperative day.

**Table 2:** Comparison of the facial nerve function according to House–Brackmann (HB) and histological findings between 40 prospective patients undergoing surgery using the Saxophone^®^ electrode (cIONM) and the last 40 patients undergoing surgery without the electrode (iIONM) prior to the prospective study

	cIONM	iIONM	*p*
Postoper. facial function			
1 day			
HB = 1	20 (50%)	28 (70%)	
6 months			
HB ≥ 2	20 (50%)	12 (30%)	0.11
HB = 1	39 (97.5%)	36 (90%)	
HB ≥ 2	1 (2.5%)	4 (10%)	0.17
Histology			
Benign	37 (92.5%)	36 (90%)	
Malignant	3 (7.5%)	4 (10%)	0.69
Sum	40	40	

**Fig. 3:**
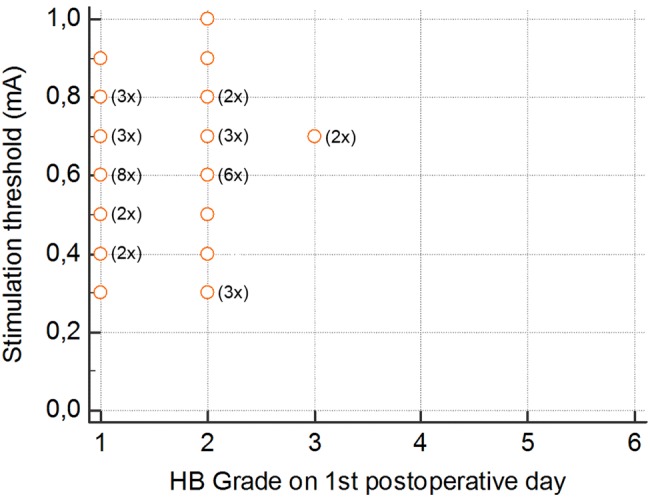
Stimulation threshold in mA relative to facial nerve function according to House–Brackmann (HB) on the first postoperative day (values for some patients overlap). No significant correlation (*p* = 0.74)

**Fig. 4:**
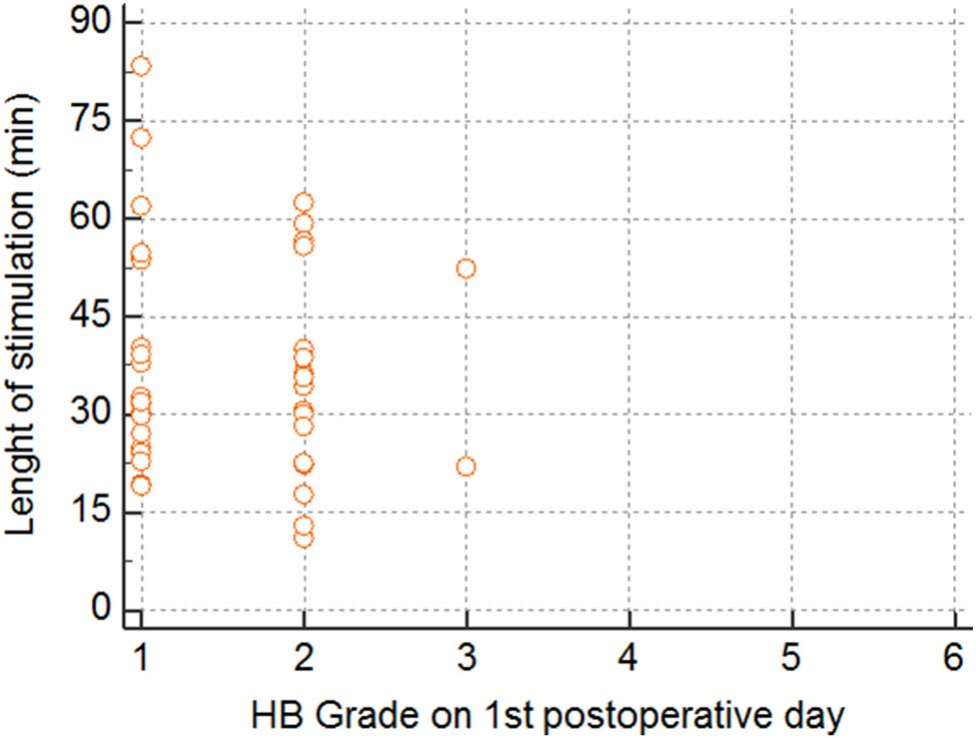
Lenght of stimulation in minutes relative to facial nerve function according to House–Brackmann (HB) on the first postoperative day (values for some patients overlap). No significant correlation (*p* = 0.51)

No statistically significant correlation between the stimulation threshold (*r* = 0.05, *p* = 0.74, 95% CI − 0.26 to 0.36, Fig. 3) or the duration of nerve stimulation and early facial paresis was found (*r* = − 0.12, *p* = 0.51, 95% CI − 0.41 to 0.21, Fig. 4).

## Discussion

Intraoperative neuromonitoring is a well-established technique in several surgical procedures which contributes to the preservation of neural structures and their functioning. Recent research has developed IONM using continuous monitoring of the nerve function, changing it from a nerve identification tool to a functional monitoring method. Thereby, hazards to the nerve, caused by stretching for example, can be detected and the surgical manipulation adapted to preserve the nerve function. This has mainly been established in thyroid surgery and in surgical procedures involving the neural structures of the cerebello-pontine angle in the posterior cranial fossa.

We have adopted this approach to present a novel method of continuous facial nerve trunk stimulation in parotidectomy. In our study, we have aimed to demonstrate the safety of this cIONM by comparing the results of a prospective study cohort to a retrospective patient group who had solely undergone intermittent IONM. cIONM of the facial nerve of up to 1 mA proved a safe procedure and was not connected to a higher rate of eFNP. Furthermore, neither the threshold of stimulation nor the length of stimulation correlated to decreased postoperative facial nerve function. The cIONM was carried out with a threshold of 0.62 ± 0.17 mA, which enabled supramaximal stimulation, and which was lower than the usual 1 mA threshold used during iIONM.

**Fig. 5:**
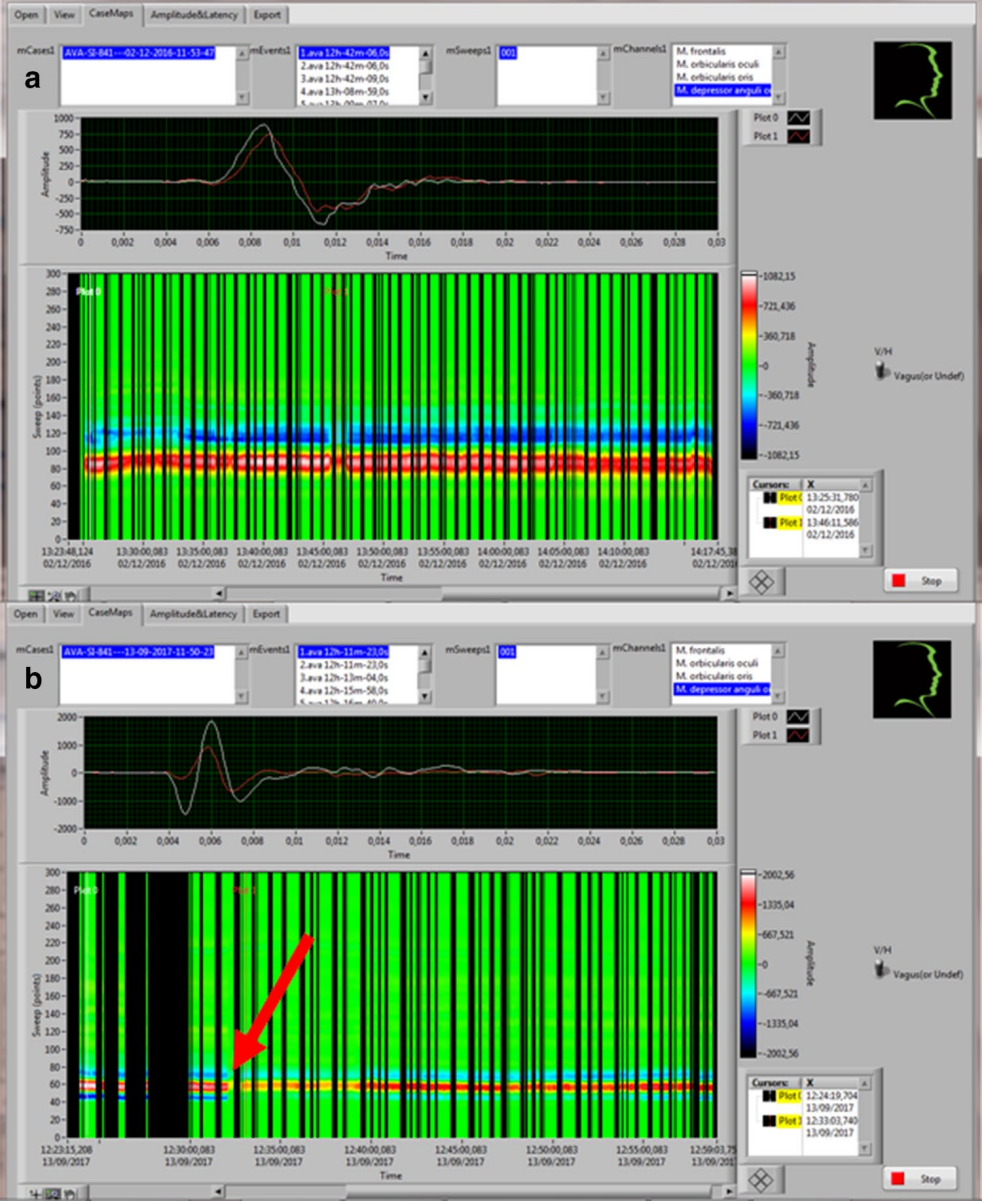
Simultaneous depiction of the amplitude of m. orbicularis oris during the entire parotidectomy: **a** stable amplitude in a patient with postoperative facial nerve function grade 1 according to House–Brackmann; **b** drop of amplitude of > 50% (red arrow) in a patient with postoperative facial nerve function grade 2 according to House–Brackmann

Bearing in mind that the amount of acquired information in amplitudes and latencies in four channel monitoring at 3 Hz for the average duration of cIONM (37.4 min) comes to over 50,000 per operation, it is clear that computerized data processing is essential for further analysis. However, scrolling through the amplitude values, it is apparent that patients with normal facial nerve function postoperatively have a stable amplitude, whereas patients with eFNP show a drop of amplitude (DOA) of > 50% (Fig. 5). No loss of signal (LOS) was noted in our study group.

Once a cut-off value for DOA and a rise in latency that correlates of eFNP is found, this could potentially be integrated into the software of the IONM device to warn the surgeon of impending nerve injury, following which the surgeon could modify the causative surgical maneuver or, for example, administer intravenous corticoids.

The postoperative facial nerve function was normal (HB1) in 50% of patients, while 45% of patients suffered a mild facial palsy (HB2) and 5% of patients had an HB3. Only one patient, who suffered from a malignant tumour, remained HB3 for 6 months postoperatively whereas all others improved to HB1. The reported rate of an eFNP of 50% was in accordance with relevant literature [1–5]. There was also no statistically significant difference when comparing the eFNP in this prospective cohort to the last 40 parotidectomies performed in our institution before the prospective study, using the same inclusion and exclusion criteria (*p* = 0.11). Although the eFNP rate in the cIONM group was higher, one should bear in mind that these patients were part of a prospective study, whereas the data for the iIONM group was collected retrospectively. It is a common finding in relevant literature that retrospective studies [11–13] tend to report lower eFNP rates when compared to prospective studies [1, 4, 5]. This inconsistent reporting of eFNP intensifies the need for objective reporting, such as computerized facial movement recognition [14, 15].

It is dissatisfactory for any surgeon conducting parotid surgery to be confronted with a patient suffering eFNP when no direct damage to the nerve occurred during dissection. That being so, it is therefore clear that intraoperative traction and stretching of the nerve play a crucial role in postoperative nerve function. This has led us to conclude that one can acquire important information on the status of the nerve by continuously stimulating the nerve and analyzing the relevant amplitudes and latencies. In our opinion, the said information will lead to a reduction in eFNP.

The application of cIONM in thyroid surgery has enabled the identification of impending nerve injury and has had a prognostic value in the postoperative palsy of the recurrent nerve [8, 9]. A DOA of > 50% together with a rise of latency of > 10% as well as complete LOS have been identified as signaling impending nerve injury. This pattern has served as a warning to surgeons in subsequent studies and has been used to modify the causative surgical maneuver in 73–82% of cases, thus reducing the postoperative palsy rate significantly [9, 16]. In a high-volume study comparing cIONM to iIONM, significantly more permanent vocal cord palsy was seen in the iIONM group [16].

Furthermore, various methods of cIONM of the facial nerve can be found in literature. These include continuous stimulation of the nerve at the root exit zone during posterior fossa surgery where a negative predictive value of a drop of amplitude of > 50% was noted [17], and transcranial multi-pulse electric stimulation of the corticobulbar pathway to stimulate the facial nerve, used by other authors in the same type of surgery. Here, cut-off values for a drop of amplitude of 50%, 35% and 0% correlated with eFNP [18]. Finally, methods of percutaneous stimulation of the facial nerve during surgery for facial vascular malformations have shown that a DOA of > 50% should serve as an alarm signal to the surgeon to stop the manipulation and to wait until the amplitude normalizes [19].

### Limitations

Due to the design of the electrode used in our study, a 360° facial trunk exposure was needed. Although the dissection of the facial nerve trunk was done meticulously using loupe lenses, in some patients this was very difficult due to the anatomy or the fact that the tumour was located directly laterally to the nerve trunk. In these cases, it is possible that unnecessary trauma was inflicted on the nerve due to the effort of implanting the electrode.

Difficulties in deriving the signals were encountered when bleeding in the wound bed or accidental electrode dislocation (1.4 per parotidectomy) occurred. In some patients, due to anatomical circumstances, electrode implantation was difficult. An average of 4.3 min was necessary for the placement. A new electrode design which enables adhesive implantation without the need to dissect the medial aspect of the nerve trunk and which is tailored to the facial nerve is needed.

In patients where fibrosis of the facial nerve trunk was present, implantation of the Saxophone^®^ electrode was not possible, as seen in one patient in our cohort.

## Conclusion

We have demonstrated the safe use of a novel method of cIONM of the facial nerve in parotid surgery. cIONM with the Saxophone^®^ electrode is a safe and easy method where neither length nor threshold of stimulation correlates to eFNP. Future development of this method could enable the recognition of impending nerve injury thus reduce eFNP.
