# Correction to: Safety of the “Saxophone^®^” electrode in parotid surgery for continuous intraoperative neuromonitoring of the facial nerve: results of a pro- and retrospective cohort study

**DOI:** 10.1007/s00405-020-05885-0

**Published:** 2020-03-06

**Authors:** Petar Stankovic, Jan Wittlinger, Robert Georgiew, Nina Dominas, Katrin Reimann, Stephan Hoch, Thomas Wilhelm, Thomas Günzel

**Affiliations:** 1 Department of Otolaryngology, Head/Neck and Facial Plastic Surgery, Sana Kliniken Leipziger Land, Rudolf-Virchow-Strasse 2, 04552 Borna, Germany; 2 Department of Otolaryngology, Head/Neck and Facial Plastic Surgery, Martin Luther University of Halle-Wittenberg, Wittenberg, Germany; 3 Department of Otolaryngology, Head/Neck and Facial Plastic Surgery, Philipps University of Marburg, Marburg, Germany; 4 Medical Faculty, Philipps University of Marburg, Marburg, Germany; 5 Department of Otolaryngology, Head and Neck Surgery, Borromaeus Hospital Leer, Leer, Germany

## Correction to: European Archives of Oto-Rhino-Laryngology 10.1007/s00405-020-05803-4

In the original publication of the article, first name and last names of all authors were swapped.

The correct author names are published in this version.

The original version was updated.

